# Clinical Evaluation of a Novel Point‐of‐Care Hematology Analyzer for Complete Blood Count With Differential

**DOI:** 10.1111/ijlh.70032

**Published:** 2025-12-08

**Authors:** Ryan C. Shean, Sterling T. Bennett

**Affiliations:** ^1^ ARUP Laboratories Salt Lake City Utah USA; ^2^ Department of Pathology University of Utah Salt Lake City Utah USA; ^3^ Department of Pathology Intermountain Medical Center Murray Utah USA

**Keywords:** CBC, hematology analyzer, method comparison, POCT, point of care

## Abstract

**Introduction:**

Point‐of‐care testing (POCT) for hematology enables clinicians to access actionable results during patient visits, supporting timely decisions such as transfusions or chemotherapy adjustments. However, POCT analyzers must demonstrate clinical performance comparable to centralized laboratory systems. We conducted a proof‐of‐concept evaluation of the HemoScreen (PixCell Medical, Israel) hematology POCT analyzer across diverse clinical settings in an integrated healthcare system.

**Methods:**

EDTA whole blood specimens from inpatient wards, emergency departments, outpatient clinics, and infusion centers underwent routine CBC testing on a Sysmex XN analyzer (Sysmex Corporation, Japan) at the central laboratory. Residual, de‐identified samples were tested within 10 min on a HemoScreen analyzer. All parameters and instrument flags were recorded.

**Results:**

From 9/15/2020 to 11/20/2020, 199 samples were analyzed, including 49 from the emergency department and 50 each from inpatient, outpatient, and infusion center settings. HGB, RBC, PLT, WBC, and absolute neutrophil count showed strong linear correlations (*R* ^2^ = 0.91–0.98) and no significant differences between instruments (*p* > 0.29). HCT and MCV values were significantly lower on HemoScreen (bias −5% and −3.6%; *p* = 0.010 and *p* < 0.001, respectively), with modestly reduced correlations (*R* ^2^ = 0.80–0.89). Neutrophil percentage was higher on HemoScreen (bias +9.0%, *p* < 0.001), whereas monocyte counts were significantly lower (bias −50%, *p* < 0.001).

**Conclusion:**

HemoScreen demonstrated generally acceptable agreement with the Sysmex XN for most parameters. However, systematic differences in HCT, MCV, neutrophil, and monocyte results warrant further investigation to assess clinical impact before broader implementation.

## 1 Introduction

One of the most important tests in the modern laboratory is the complete blood count (CBC). The CBC is included in general health screening panels and can be used to diagnose a variety of diseases and conditions including anemia, infection, inflammation, bleeding disorders, leukemia, and others [1]. The CBC is also used to monitor the status of diseases, the effectiveness of treatments, and the safety of treatments known to adversely affect blood cells, such as chemotherapy and radiation. The CBC is utilized across almost every medical specialty and every practice setting. As such, the CBC is the most commonly ordered laboratory test in the world [2] and in our multi‐state integrated health care system. The underlying technology of hematology analyzers has significantly advanced since the days of manual counts with a hemocytometer [3]. Modern instruments are extremely automated and have high throughput, laboratory information system integration, and expanded flagging parameters. Additionally, many recently developed technologies have allowed dramatic miniaturization, making near‐patient hematology analyzers a reality [4, 5].

Point‐of‐care testing (POCT) has many upsides. Given the utility and frequency that CBC results impact management decisions, having patient results at bedside or during an office visit can improve patient care by reducing turnaround time (TAT), streamlining care, improving physician workflows, and providing laboratory answers to patients during their office visits. However, centralized instrumentation and expertise also have many upsides and allow high throughput testing, concentrated skills for technical staff, centralized training, and economy of scale in purchasing and other laboratory operations, which can dramatically reduce costs compared to POCT testing. Despite the advantages of centralized testing, certain patient populations and practice settings would likely benefit from having near‐patient CBC testing, such as monitoring blood counts in clozapine patients [6, 7] and in hematology/oncology infusion centers where decisions regarding blood product transfusions and toxic chemotherapy regimens are made. Rapid availability of CBC results would allow these important decisions to be made during the patient's office visit or remove the need for patients to present ahead of time to get labs drawn, a particularly onerous task for patients who live far away from where their health care is delivered. However, despite widely available POCT methods for coagulation, blood chemistry, and hemoglobin analysis, the availability of fully featured POCT hematology analysis has lagged behind [8]. As a result of this documented and defined unmet need [9] there has been industry interest in developing POCT CBC devices, a variety of which are currently available in the United States and European Union with a range of different sample types, test methodologies, reporting options, turnaround times, and compliance categories for these devices. (Table 1) [10, 11, 12, 13, 14].

**TABLE 1:** Point‐of‐care CBC analyzers available in the United States.

Parameter	Analyzer							
	PixCell HemoScreen	Sight OLO	Sysmex pocH‐100i	Sysmex XW‐100	Sysmex XQ‐320	QBC Star	HORIBA Microsemi CRP LC‐767G	Beckman coulter DxH 500
Regulatory status	CE, CLIA moderate complexity	CE, CLIA moderate complexity	CE, CLIA moderate complexity	CE, CLIA waived	CE, CLIA moderate complexity	CE, CLIA moderate complexity	CE mark	CE Mark, CLIA moderate complexity
Sample type	Venous whole blood or capillary finger stick	Capillary finger stick or venous whole blood	Whole blood	Venous whole blood	Whole blood	Finger stick or venous whole blood	Capillary and venous whole blood	Capillary and venous whole blood
Published TAT	5 min	~10 min	2.4 min	3 min	~1 min	7 min	1 min	1 min
Methodology	Digital image analysis	Digital image analysis	Impedance/flow cytometry	Impedance/flow cytometry	Impedance/flow cytometry	Centrifugal analysis	Impedance/flow cytometry	Impedance/flow cytometry
WBC	X	X	X	X	X	X	X	X
RBC	X	X	X	X	X		X	X
HGB	X	X	X	X	X	X	X	X
HCT	X	X	X	X	X	X	X	X
MCV	X	X	X	X	X		X	X
MCH	X	X	X	X	X		X	X
MCHC	X	X	X	X	X	X	X	X
RDW	X	X	X	X	X		X	X
PLT	X	X	X	X	X	X	X	X
MPV	X				X		X	X
NRBC	X	Flag						
IG # & %	Flag	Flag						
NEU # & %	X	X	X	X	X			X
LYM # & %	X	X	X	X			X	X
MON # & %	X	X					X	X
EOS # & %	X	X						X
BAS # & %	X	X						X
Mixed # & % (MON, EOS, BAS)			X	X	X			
Gran # & % (NEU, EOS, BAS)						X	X	
LYM + MON # & %						X		
Limitations	Not intended for use in children under 2 years old	Not intended for use in infants under 3 months old		Not intended for use in critical care, chronic illness, oncology, or children under 2 years old				

One relatively new POCT testing device, which has been FDA cleared for moderate complexity testing, is the PixCell HemoScreen analyzer (PixCell Medical, Israel). This device uses viscoelastic focusing to create a single‐file flow of cells with much lower fluid volumes than traditional hydrodynamic focusing. This miniaturization allows a desktop footprint device to produce a CBC including a 5‐part differential and platelet count. Previous studies across a variety of settings have shown generally acceptable agreement with leading core laboratory analyzers and other POCT devices [15, 16, 17, 18].

Because patient populations, practice settings, and many other pre‐analytic variables can significantly change analytic performance [19], the aim of this project was to conduct a real‐world evaluation of the HemoScreen POCT device compared to our core laboratory Sysmex XN (Sysmex, Japan) hematology analyzers across a variety of practice settings, including the inpatient units, emergency department, outpatient clinics, and outpatient infusion centers.

## 2 Materials and Methods

Between 9/15/2020 and 11/20/2020, a total of 199 clinical samples sent for a CBC at the central laboratory as part of routine clinical care were selected from four clinical settings: inpatient units, an infusion center, and the emergency department at Intermountain Medical Center, Murray, Utah, and outpatient clinics that refer specimens to the Intermountain Central Laboratory on the Intermountain Medical Center campus. Equal numbers of samples were randomly selected for the project from the four clinical settings before clinical CBC testing was completed. Residual blood from each sample was de‐identified and routed for POCT testing.

Clinical CBC results were generated on one of three Sysmex XN analyzers according to laboratory standard operating procedures in accordance with manufacturer's instructions. Within 10 min of Sysmex testing, the residual specimens were tested on one of two HemoScreen analyzers. HemoScreen setup, verification, and testing were performed according to manufacturer's instructions. Although three different Sysmex instruments and two different HemoScreen analyzers were used for this project, each specimen was tested on only one of each.

HemoScreen flags that require further review according to the manufacturer's instructions include: (1) *, abnormal cells with results displayed, (2) ABN, abnormal cells with results suppressed, (3) !, platelet clumps, (4) ~, abnormal distribution of platelet cell volumes, and (5) ^, WBC < 2.0 × 10^3^/μL but within the reportable range. Specimens with one or more of these flags had a blood smear prepared and examined with a 100 cells WBC differential count. For each flagged specimen, the percent of each WBC subpopulation from the HemoScreen automated differential was compared against the 99% binomial proportion confidence interval of the corresponding manual differential result. A significance level of 0.01 was used to account for multiple comparisons with each differential. Any automated differential result outside of the confidence interval of its corresponding manual differential result was considered statistically significantly different [20, 21, 22].

Results were analyzed in R v4.2 [23]. Additional subgroup analysis was performed by delineating results based on specific analyzer pairs and patient type (inpatient, outpatient, infusion center, or emergency department). Linear correlation was assessed by Pearson's correlation and visualized with linear regression. Bias was assessed using difference of means and visualized with Bland Altman plots. Precision was measured using daily low, normal, and high control materials and was calculated using standard deviation and coefficient of variation. Statistical significance between instrument values was calculated using paired *t*‐tests. The predefined significance threshold was set at *p* < 0.05 with Bonferroni correction when multiple tests were performed at once. Clinically significant differences were adjudicated by the authors' expert opinion.

This project has been approved by the Intermountain Health Institutional Review Board IRB# 105330.

## 3 Results

Between 9/15/2020 and 11/20/2020, a total of 199 samples were tested on both the HemoScreen and Sysmex platforms, including 49 samples from the emergency department and 50 samples each from the inpatient units, outpatient clinics, and infusion center. The average time difference between Sysmex and HemoScreen testing was 2.3 min. There were two samples from the emergency department and one from the infusion center that failed to generate results on the HemoScreen and were excluded from further analysis.

Between‐day imprecision for the HemoScreen instrument is shown in Table S1. Coefficients of variation (CVs) for total WBC count ranged from 7.4% to 7.8%, whereas HGB CVs varied from 1.8% to 4.4%. PLT CVs fell between 2.8% and 4.8%, and absolute neutrophil counts showed CVs between 9.1% and 10.1%.

**FIGURE 1:**
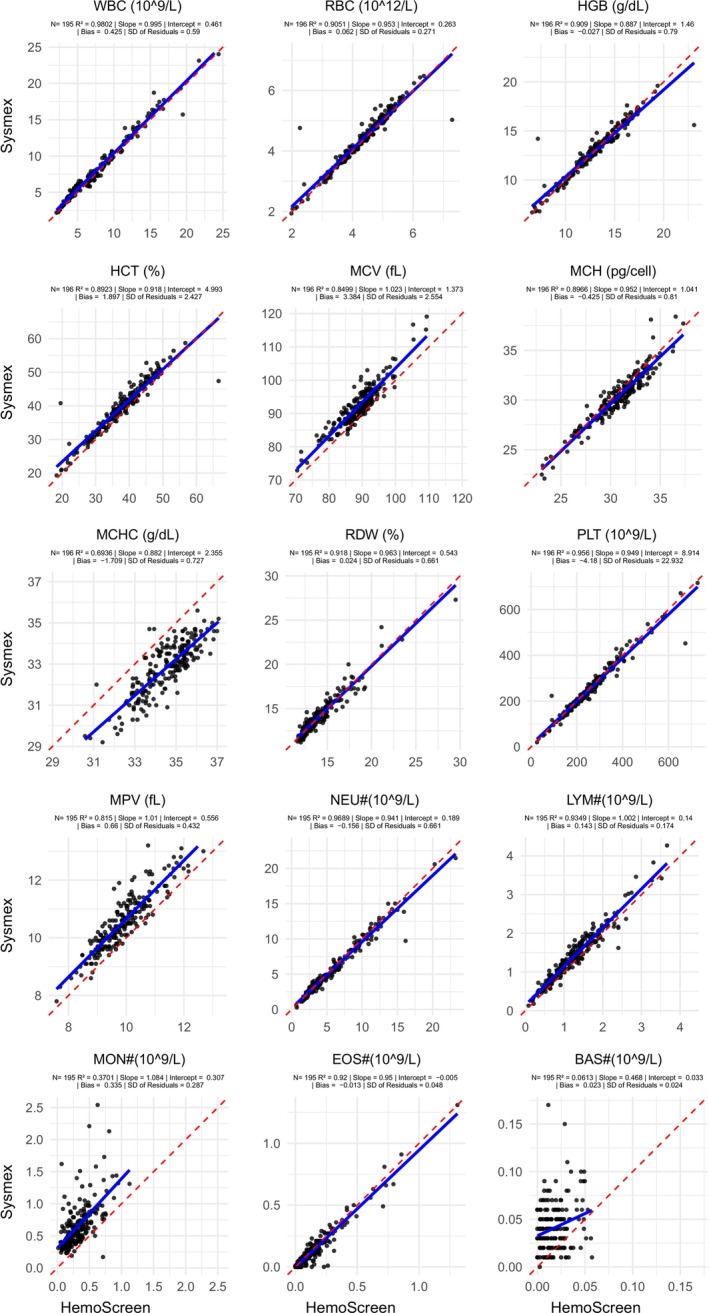
All sites and analyzers pooled scatter plots: CBC parameter and units are displayed above each subplot. HemoScreen values are plotted on the *X*‐axis and Sysmex values are plotted on the *Y*‐axis. Each dot represents one sample tested on both instruments. Blue line is linear regression line. Red dotted line is *X* = *Y* and represents perfect correlation. Total number of samples co‐tested, linear regression slope and intercept, *R* ^2^, bias, and SD of residuals are displayed below each parameter for the corresponding plot.

**FIGURE 2:**
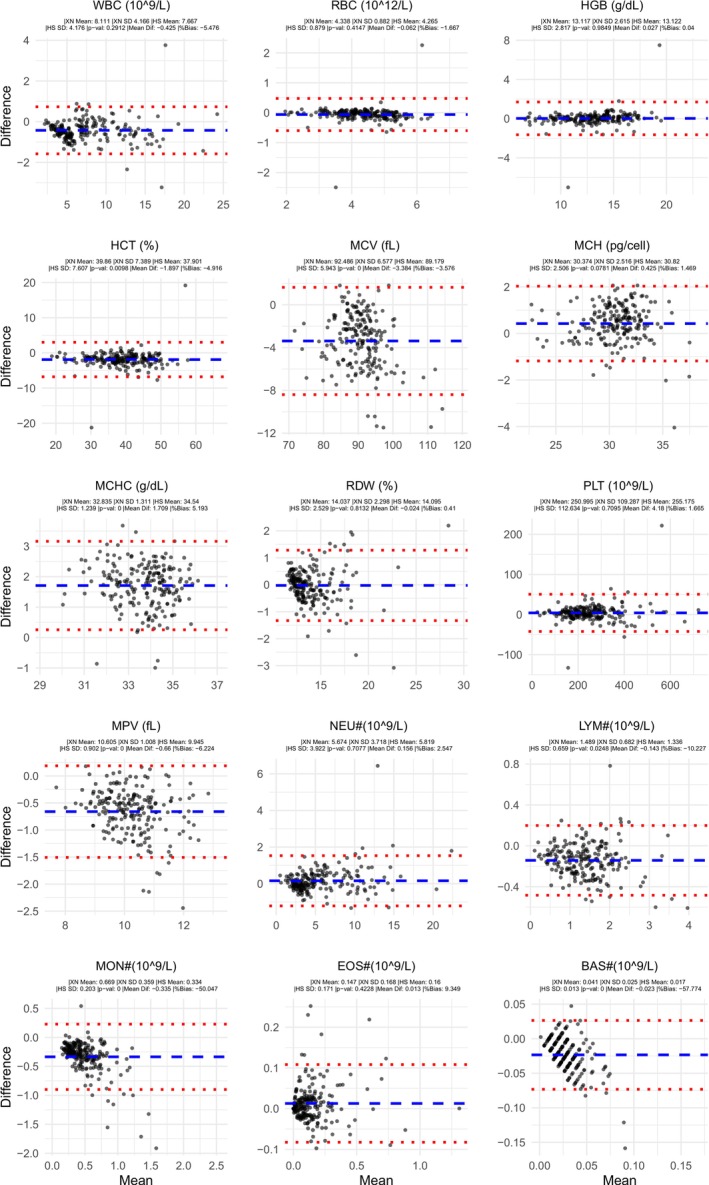
All site and analyzers pooled Bland–Altman plots: CBC parameter and units are displayed above each subplot. Average value for the two analyzers is plotted on the *X*‐axis and difference between the HemoScreen instrument minus the Sysmex XN instrument is plotted on the *Y*‐axis. Blue dashed line represents the mean difference between HemoScreen and Sysmex. Red dotted lines represent the upper and lower limits of agreement (mean difference ± 1.96 * standard deviation of difference). Individual means, standard deviations, *p* value from a paired sample *t*‐test of the values, overall mean difference and bias are shown in the subheading for each subplot.

Overall, pooled data from all analyzers showed generally acceptable agreement (Figures 1 and 2, Table 2). WBC, RBC, HGB, MCH, RDW, PLT, absolute neutrophil count (NEU#; often written as ANC), absolute eosinophil count (EOS#), lymphocyte percentage (LYM%), and eosinophil percentage (EOS%) showed high correlation and statistically insignificant bias (*p* > 0.05). HCT, MCV, MPV, absolute lymphocyte count (LYM#), and neutrophil percentage (NEU%) showed high correlation, but with statistically significant bias (*p* < 0.05). MCHC, absolute monocyte count (MON#), and monocyte percentage (MON%) showed moderate correlation, and absolute basophil count (BAS#) and basophil percentage (BAS%) showed low correlation, all with statistically significant bias (*p* < 0.05). Of those parameters with statistically significant bias, only MON# bias was judged to be potentially clinically significant.

**TABLE 2:** Intermountain Health—HemoScreen Versus Sysmex XN.

Parameter	*N*	*R* ^2^	Slope	Intercept	SD_residuals_	Sysmex		HemoScreen		*t‐*test *p*	Mean difference	
						Mean	SD	Mean	SD		Absolute	Percent
WBC (10^9^/L)	195	0.980	0.995	0.461	0.590	8.11	4.17	7.67	4.18	0.291	−0.43	−5.5%
RBC (10^12^/L)	196	0.950	0.953	0.263	0.271	4.34	0.88	4.27	0.79	0.415	−0.06	−1.7%
HGB (g/dL)	196	0.909	0.887	1.460	0.790	13.12	2.62	13.12	2.82	0.985	0.03	0.0%
HCT (%)	196	0.892	0.918	4.993	2.427	39.86	7.39	37.90	7.61	0.010	−1.90	−4.9%
MCV (fL)	196	0.850	1.023	1.373	2.554	92.49	6.58	89.18	5.94	0.000	−3.38	−3.6%
MCH (pg)	196	0.897	0.952	1.041	0.810	30.37	2.52	30.82	2.51	0.078	0.43	1.5%
MCHC g/dL	196	0.694	0.882	2.355	0.727	32.84	1.31	34.54	1.24	0.000	1.71	5.2%
RDW (%)	195	0.918	0.963	0.543	0.661	14.04	2.30	14.10	2.53	0.813	−0.02	−0.4%
PLT (10^9^/L)	196	0.956	0.949	8.914	22.932	251.00	109.29	255.18	112.63	0.710	4.18	1.7%
MPV (fL)	195	0.815	1.010	0.556	0.432	10.61	1.01	9.95	0.90	0.000	−0.66	−6.2%
NEU# (10^9^/L)	195	0.969	0.941	0.189	0.661	5.67	3.72	5.82	3.92	0.708	0.16	2.5%
LYM# (10^9^/L)	195	0.935	1.002	0.140	0.174	1.49	0.68	1.34	0.66	0.025	−0.14	−10.2%
MON# (10^9^/L)	195	0.370	1.084	0.307	0.287	0.67	0.36	0.33	0.20	0.000	−0.34	−50.0%
EOS# (10^9^/L)	195	0.920	0.950	−0.005	0.048	0.15	0.17	0.16	0.17	0.423	0.01	9.3%
BAS# (10^9^/L)	195	0.061	0.468	0.033	0.024	0.04	0.03	0.02	0.01	0.000	0.02	−57.8%
NEU%	195	0.850	0.943	−1.860	4.852	66.04	12.42	71.98	12.26	0.000	5.99	9.0%
LYM%	195	0.962	0.991	1.063	2.054	21.16	10.45	20.27	10.45	0.398	−0.88	−4.2%
MON%	195	0.441	0.987	4.299	3.215	9.13	4.29	4.92	2.89	0.000	−4.24	−46.1%
EOS%	195	0.917	0.900	−0.067	0.690	2.21	2.38	2.55	2.55	0.169	0.32	15.5%
BAS%	195	0.029	0.207	0.509	0.330	0.57	0.34	0.28	0.27	0.000	−0.29	−51.2%

Forty specimens (20%) were flagged by HemoScreen for further review: 32 with the * flag (abnormal cells), 3 with the ! flag (platelet clumps), 4 with both ! and * flags (platelet clumps and abnormal cells), and 1 with both ! and ABN flags (platelet clumps and abnormal cells with suppressed differential). No ~ or ^ flags were generated. Thirty‐eight of the specimens had blood smears prepared and examined. Of the 30 specimens with an abnormal cells flag, abnormal cells were observed on 14 blood smears (47%). Of the 8 specimens with a platelet clumps flag, platelet clumps were observed on 3 blood smears (38%). Of the 37 specimens with both an automated differential and a manual differential, statistically significant differences were observed in one or more WBC subpopulations in 15 (41%). Differences consisted of higher automated neutrophil counts or lower automated lymphocyte, monocyte, or basophil counts. Additional details are provided in the supplemental data Table S2. As there were two different HemoScreen instruments and three different Sysmex XN instruments used in this project, subgroup analysis by specific analyzer pairs was also performed (Table S1). For the selected analytes examined (WBC, HGB, RBC, PLT, and NEU#), the average absolute mean difference and bias between instrument pairs was similar to the overall mean difference and bias for the main dataset. No differences between analyzer pairs were statistically significant (*p* > 0.05) by one‐way ANOVA.

Subgroup analysis by clinical setting of patient samples was also performed for the emergency department (Figures S1 and S2), outpatient clinics (Figures S3 and S4), inpatient wards (Figures S5 and S6), and infusion center (Figures S7 and S8). Although the underlying distribution of data was different between clinical settings, the mean difference and bias between Sysmex XN and HemoScreen analyzers were not significantly different by one‐way ANOVA (*p* > 0.05).

## 4 Discussion

Overall, we report acceptable agreement for the HemoScreen and Sysmex XN analyzers, mirroring results of previous studies comparing the two instruments [15, 16, 17, 18, 24, 25] (Table 3), even in a real‐world setting. Importantly, in the literature and this project, WBC, RBC, HGB, PLT, and NEU#, the clinically most meaningful parameters of the CBC, showed high concordance. All other parameters had adequate concordance, except monocyte and basophil counts correlated poorly. With only a few exceptions, basophil counts were higher on Sysmex than HemoScreen, although counts were extremely low, and differences were of doubtful clinical importance in most cases. Monocyte differences generally appeared more clinically relevant in both this project and the literature unless reported with HemoScreen‐specific reference intervals, suggesting that in settings where serial monocyte counts may be required (chronic myelomonocytic leukemia, hairy cell leukemia, etc.) HemoScreen and Sysmex results should not be considered interchangeable.

**TABLE 3:** HemoScreen versus Sysmex XN—intermountain results versus published data.

Parameter	*N*	*R* ^2^	*R* ^2^	*R*	*R*	*R* ^2^	*R* ^2^	*R* ^2^	Bias	Bias	Bias	Bias	Bias	Bias	Bias
Reference		Intermountain	15	16	17	18	21	22	Intermountain	15	16	17	18	21	22
WBC (10^9^/L)	195	0.980		0.981	0.997	0.916	0.995	0.960	−5.50%		−0.43	−6.15%	−7.20%	0.07	0.44
RBC (10^12^/L)	196	0.950		0.974	0.987	0.950	0.993		−1.22%	−0.40%	−0.05	2.33%		−0.11	0.04
HGB (g/dL)	196	0.909		0.964	0.978	0.956			0.19%	−0.30%	0.02	1.56%		−4.43	0.20
HCT (%)	196	0.892		0.958					−4.91%	−0.50%	−1.91	5.26%			−0.02
MCV (fL)	196	0.850	0.903	0.946		0.965			−3.73%	0.08%	−3.39	2.25%			−5.30
MCH (pg)	196	0.897	0.916	0.939		0.909			1.46%	0.15%	0.45	0.00%			−0.12
MCHC g/dL	196	0.694		0.870		0.608			5.07%		1.71	−3.86%			17.20
RDW (%)	195	0.918				0.894			0.05%		0.01	−2.17%			
PLT (10^9^/L)	196	0.956	0.918	0.964	0.991	0.956	0.994		1.50%	−4.20%	3.76	1.56%		19.60	17.20
MPV (fL)	195	0.815							−6.45%		−0.66				
NEU# (10^9^/L)	195	0.969			0.993	0.924		0.960	2.22%		0.13	−12.90%	−8.30%	−0.14	
LYM# (10^9^/L)	195	0.935				0.975			−10.41%		−0.15	−5.56%			
MON# (10^9^/L)	195	0.370				0.753			−65.61%		−0.33	30.20%			
EOS# (10^9^/L)	195	0.920				0.928		0.940	5.68%		0.01	−17.60%	−10.50%		
BAS# (10^9^/L)	195	0.061				0.452			−80.57%		−0.02	50.00%			

The significant differences observed for MCV, MCH, MCHC, and MPV are largely explainable by differences in analytic methodology. The HemoScreen methodology for calculating MCV and MPV differs from impedance‐based methods such as those used on the Sysmex instrument. Instead of correlating electric impedance to volume, the HemoScreen device measures the MCV and MPV from the geometry of cell images [15]. Some variance is also noted for HCT because the HemoScreen calculates this parameter from MCV and RBC count rather than as a sum of the individual RBC volume measurements on Sysmex. MCH is directly measured on the HemoScreen using a per‐cell spectral absorbance method, whereas the Sysmex XN device calculates MCH from HGB and RBC count. There is no universal gold standard for MCH measurements, so variance in this parameter is expected, and further work is needed to standardize values across instruments. Given that MCHC is calculated from the MCH and MCV, there are also expected differences in MCHC between the two instruments. Interestingly, this study showed less correlation of MCV between PixCell and Sysmex XN instruments than previously published, perhaps due to the more heterogenous nature of samples. Regardless, if precise measurements near clinically important decision thresholds are required for MCV or MCHC, the use of a core laboratory analyzer is still recommended.

The HemoScreen is configured to flag specimens for suspected abnormal cells (NRBCs, immature granulocytes, blasts, atypical lymphocytes, and band forms), platelet clumps, and an abnormal distribution of platelet volumes. In addition, specimens with very low WBC counts are flagged for review. In our results, 38% of cases flagged for possible platelet clumps had platelet clumps observed on the blood smears, and 47% of the cases flagged for possible abnormal cells had abnormal cells observed on the blood smears. A minority of cases (41%) had one or more statistically significant differences between the automated and manual differentials in the form of higher neutrophils, lower lymphocytes, lower monocytes, and/or lower basophils from the HemoScreen. With a slight majority of flagged cases having no observed corresponding abnormalities on blood smears, our results suggest that HemoScreen flagging criteria are set conservatively to minimize the risk of missing clinically important findings. Over time, laboratories can evaluate the results of cases flagged for further review to determine thresholds for specific actions such as examining smears or performing manual differentials.

Overall, the HemoScreen instrument is less precise than would be expected from a main laboratory analyzer. Some literature has reported limits of acceptable imprecision based on the current state of the art (WBC CV 1.5%–6%; HGB CV 0.9%–1.43%; PLT CV 3%–5%; NEU# 2.5%–10%) [26]. The average precision values on the HemoScreen (WBC CV 7.4%–7.8%; HGB CV 1.8%–4.4%; PLT CV 2.81%–4.78%; NEU# CV 9.1%–10.4%) slightly exceeded these values for all parameters except platelet count. This underscores the important point that POCT is almost always less precise and less accurate than core lab testing, so extreme care should be taken during validation and implementation planning to ensure that this variability will not negatively impact patient care.

Furthermore, samples occasionally failed on the HemoScreen analyzer for unknown reasons, which could present an issue for a standalone device. Although flagging rates were briefly compared in this study, a comprehensive verification of flags and thresholds for manual review would need to be undertaken before clinical implementation.

Limitations of this project include the moderate sample size, which limits the power for subgroup analysis. Additional limitations include lack of pre‐selected samples that fill the entire analytic measurement range or allow focused analysis near clinical decision points. Additionally, pre‐defined power calculations were not performed, and the sample size was limited by practical considerations. Further limitations include lack of operational data collection, such as TAT, cost, or clinical setting workflow impacts. Clinical significance was also determined by expert opinion. As such, this study doesn't represent a clinical validation of the scale that would be required to bring on a new instrument. In this project, trained laboratory staff conducted the POCT testing procedure. Subsequently, non‐laboratory staff have successfully operated the device in more than 20 locations in our health system.

A principal strength of this project is the novel use of real‐world samples from a diverse range of practice settings, including inpatient wards, outpatient clinics, an emergency department, and an infusion center. As a result, practice settings include both those frequently studied for POCT use, as well as other novel settings. In addition, tests were run in real‐world fashion, with samples tested only once on only one of multiple possible analyzers for each platform. The real‐world configuration of this project means its results are likely more generalizable than results from tightly controlled studies in contrived environments.

Strengths of the HemoScreen device include its compact design and low counter‐top footprint, cartridge‐based testing, integrated quality controls, maintenance/QC lockouts, integration with some commercial middleware systems, and potential to reduce TAT in a variety of practice settings. Suitable clinical settings may include infusion centers, urgent care clinics, and long‐distance or rural outpatient settings.

Weaknesses of the device include its FDA moderate‐complexity categorization with associated training and competency assessment requirements. Increased per‐sample cost of POCT compared to core lab results may also be a significant limitation depending on the scenario. Less suitable clinical settings may include settings where core lab CBC information is readily available or where short TAT does not alter patient management because decisions will be delayed until other test results are returned from the core lab. In our case, although CBC TAT would have improved by implementing POCT CBC testing in our emergency department and infusion center, clinicians would still wait for chemistry results from the core lab before making key clinical decisions. For this reason, the decision was made not to implement POCT CBC testing in our emergency department or infusion center; however, on the strength of this project, POCT CBC testing has been deployed in multiple urgent care clinics.

In conclusion, the HemoScreen device delivers rapid CBC results with sufficient accuracy for many clinical scenarios. Its analytical performance makes it a candidate platform when POCT hematology testing is appropriate in the context of other factors, including cost, regulatory compliance, workflow, concurrently ordered tests, and other constraints.

## Author Contributions

All authors designed, analyzed, and wrote the final manuscript.

## Funding

The authors have nothing to report.

## Consent

This project has been approved by the Intermountain Health Institutional Review Board IRB# 105330; samples were de‐identified and consent was not required.

## Conflicts of Interest

The authors declare no conflicts of interest.

## Supporting information


**Data S1:** ijlh70032‐sup‐0001‐supinfo.zip.

**Table S1:** Between day imprecision on the HemoScreen instrument.
**Table S2:** Hemoscreen flagging analysis.

## Data Availability

The data that support the findings of this study are available from the corresponding author upon reasonable request.
