# Supplementary figures and images for: Clinical Evaluation of a Novel Point‐of‐Care Hematology Analyzer for Complete Blood Count With Differential
Source: Int J Lab Hematol. 2025 Dec 8;48(2):353–64. doi: 10.1111/ijlh.70032 (PMC12956494; doi:10.1111/ijlh.70032)

Figure S1 - Emergency Department Bland-Altman Plots

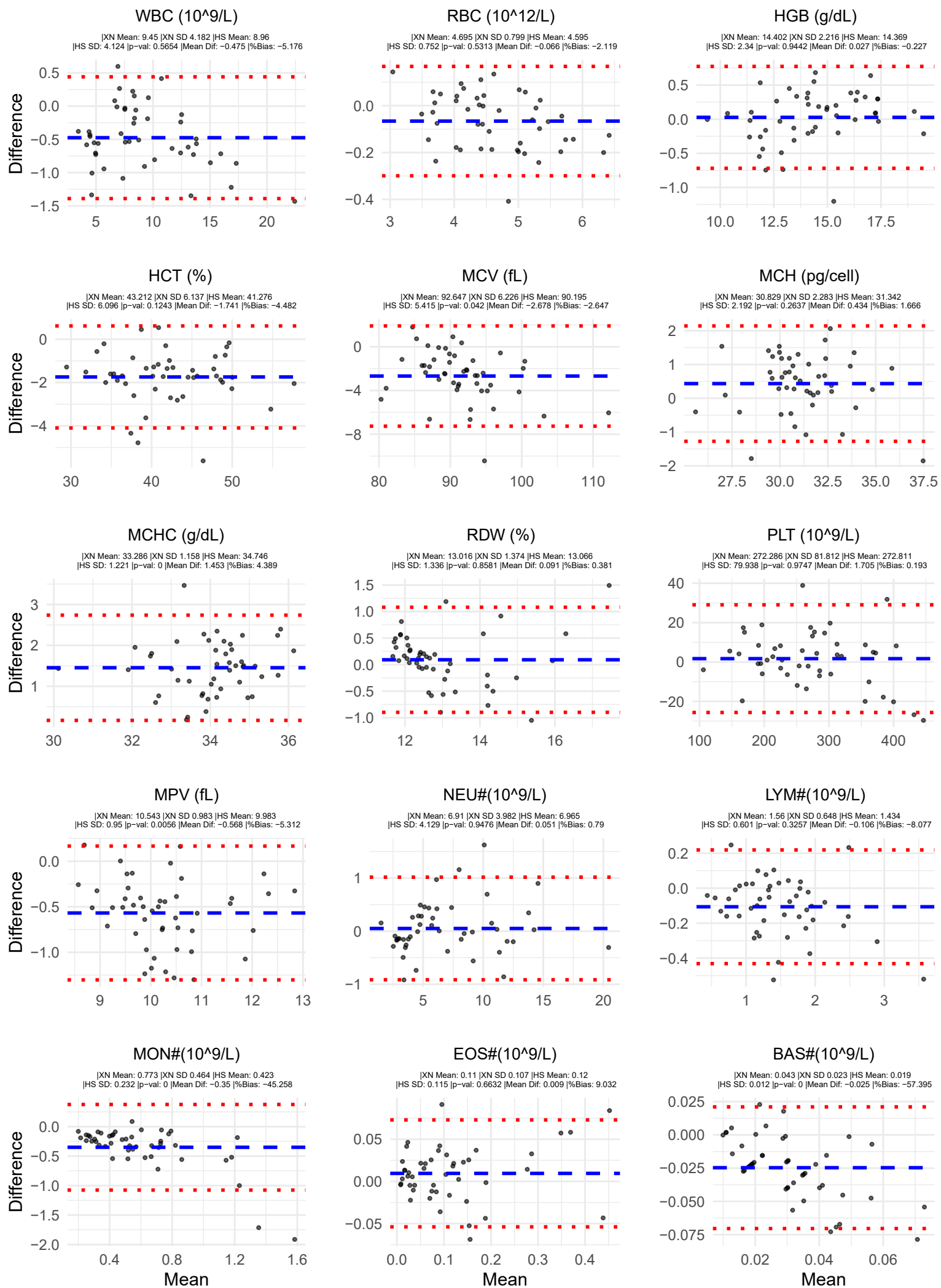

Supplement: Supplementary file 1 — Data S1: ijlh70032‐sup‐0001‐supinfo.zip. [file IJLH-48-353-s002.zip › Supplementary Material/Figure S1.pdf]

Figure S2 - Emergency Department Scatter Plots

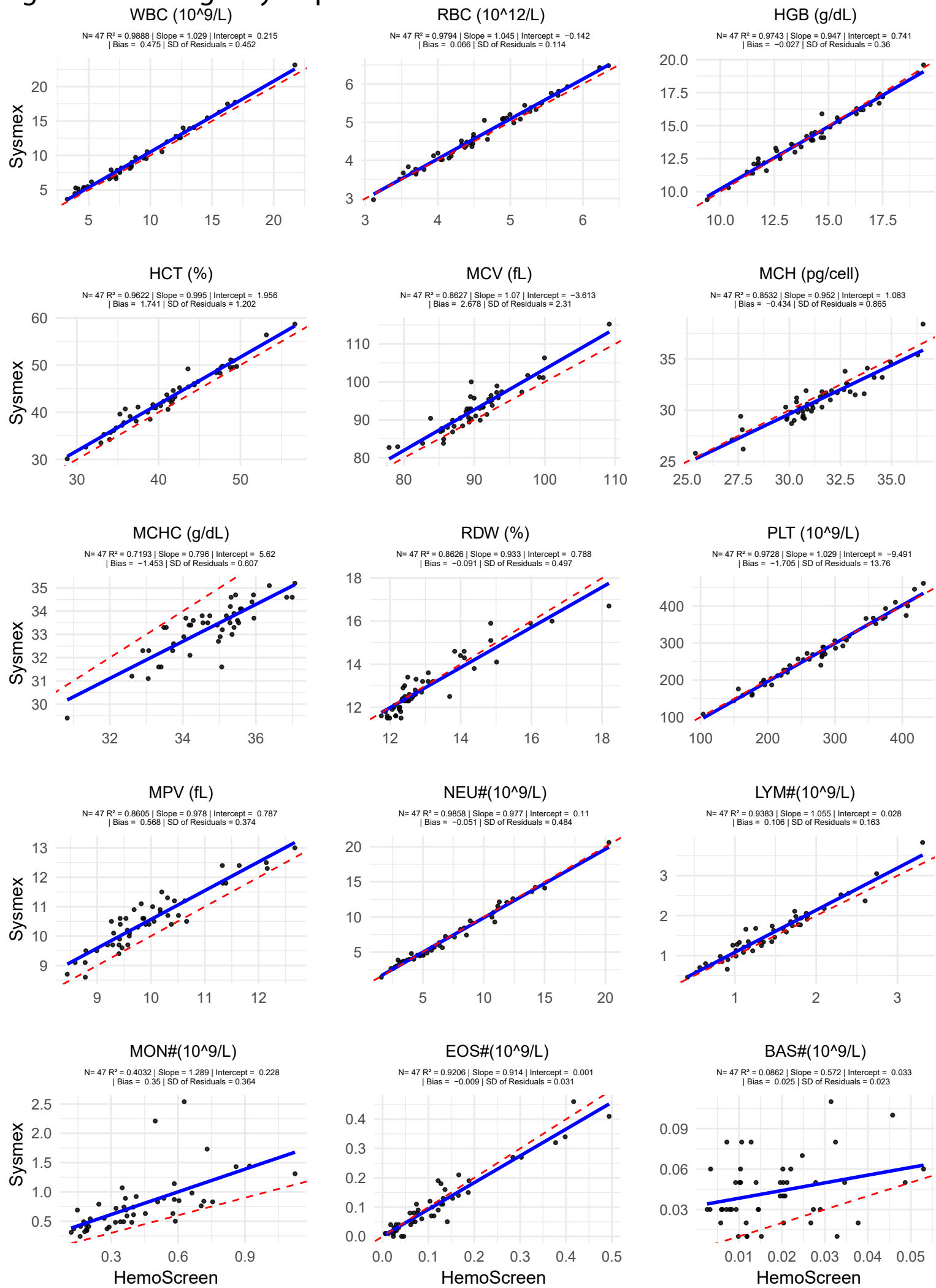

Supplement: Supplementary file 1 — Data S1: ijlh70032‐sup‐0001‐supinfo.zip. [file IJLH-48-353-s002.zip › Supplementary Material/Figure S2.pdf]

Figure S3 - Outpatient Bland-Altman Plots

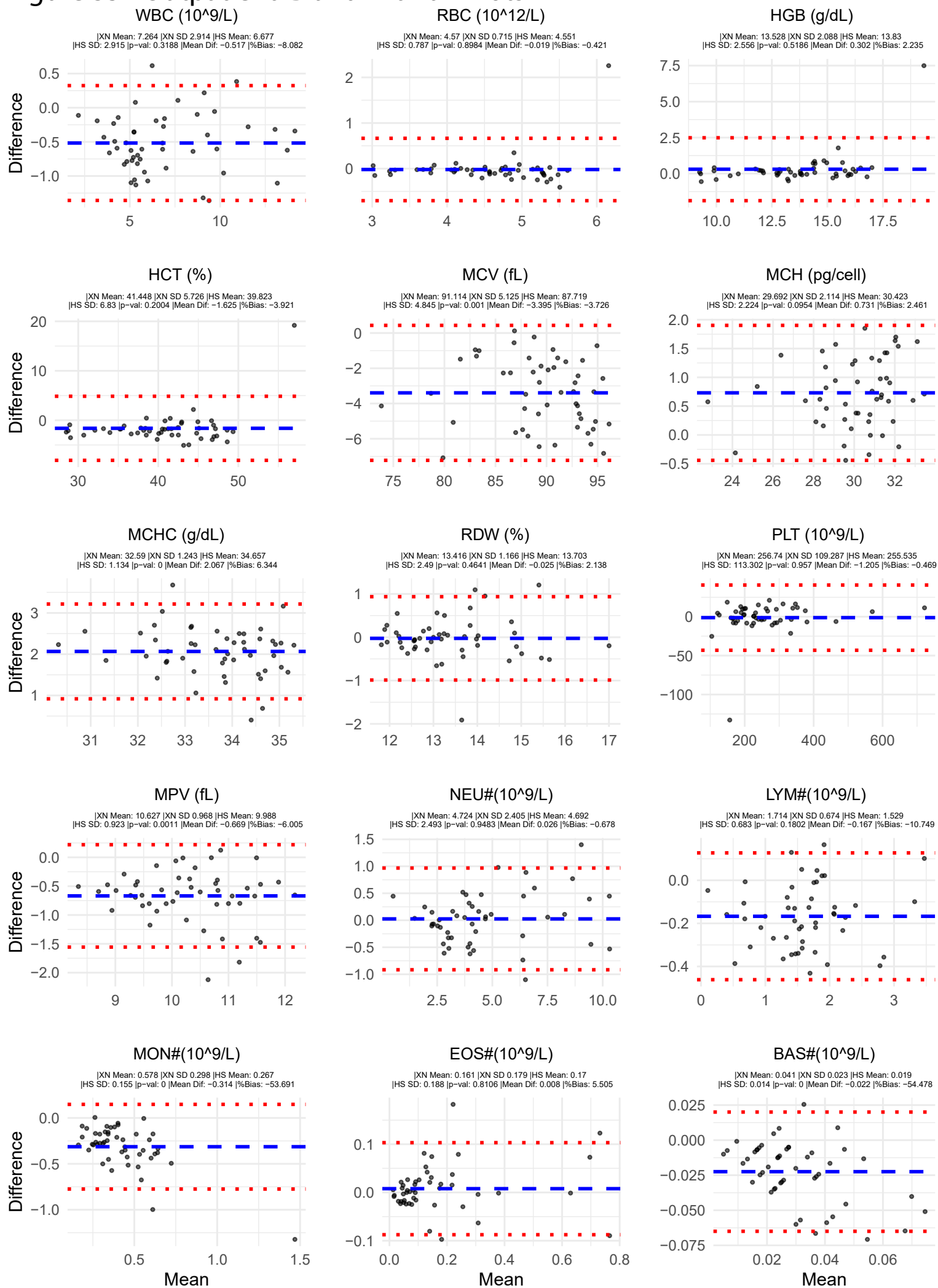

Supplement: Supplementary file 1 — Data S1: ijlh70032‐sup‐0001‐supinfo.zip. [file IJLH-48-353-s002.zip › Supplementary Material/Figure S3.pdf]

Figure S4 - Outpatient Scatter Plots

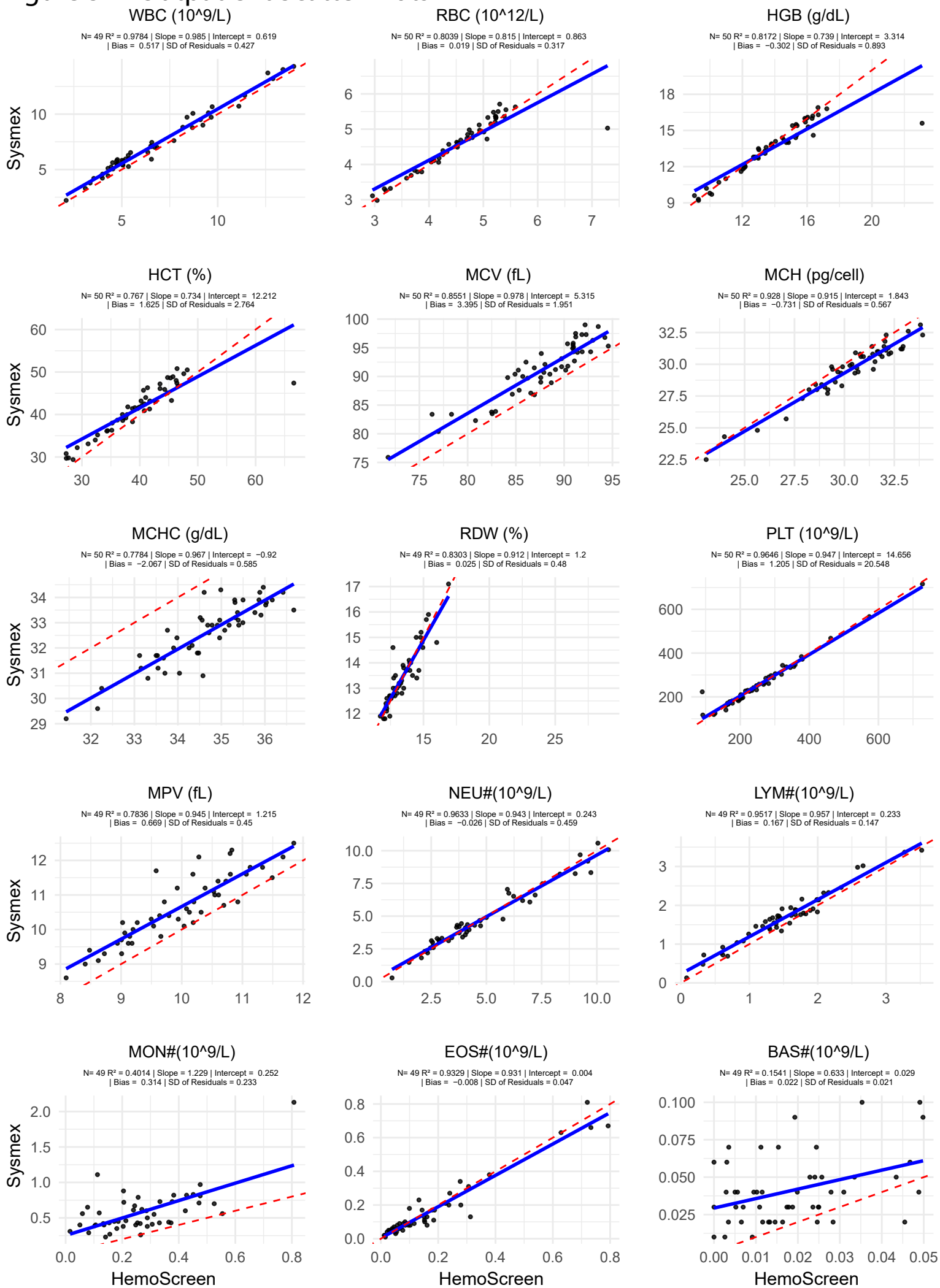

Supplement: Supplementary file 1 — Data S1: ijlh70032‐sup‐0001‐supinfo.zip. [file IJLH-48-353-s002.zip › Supplementary Material/Figure S4.pdf]

Figure S5 - Inpatient Bland-Altman Plots

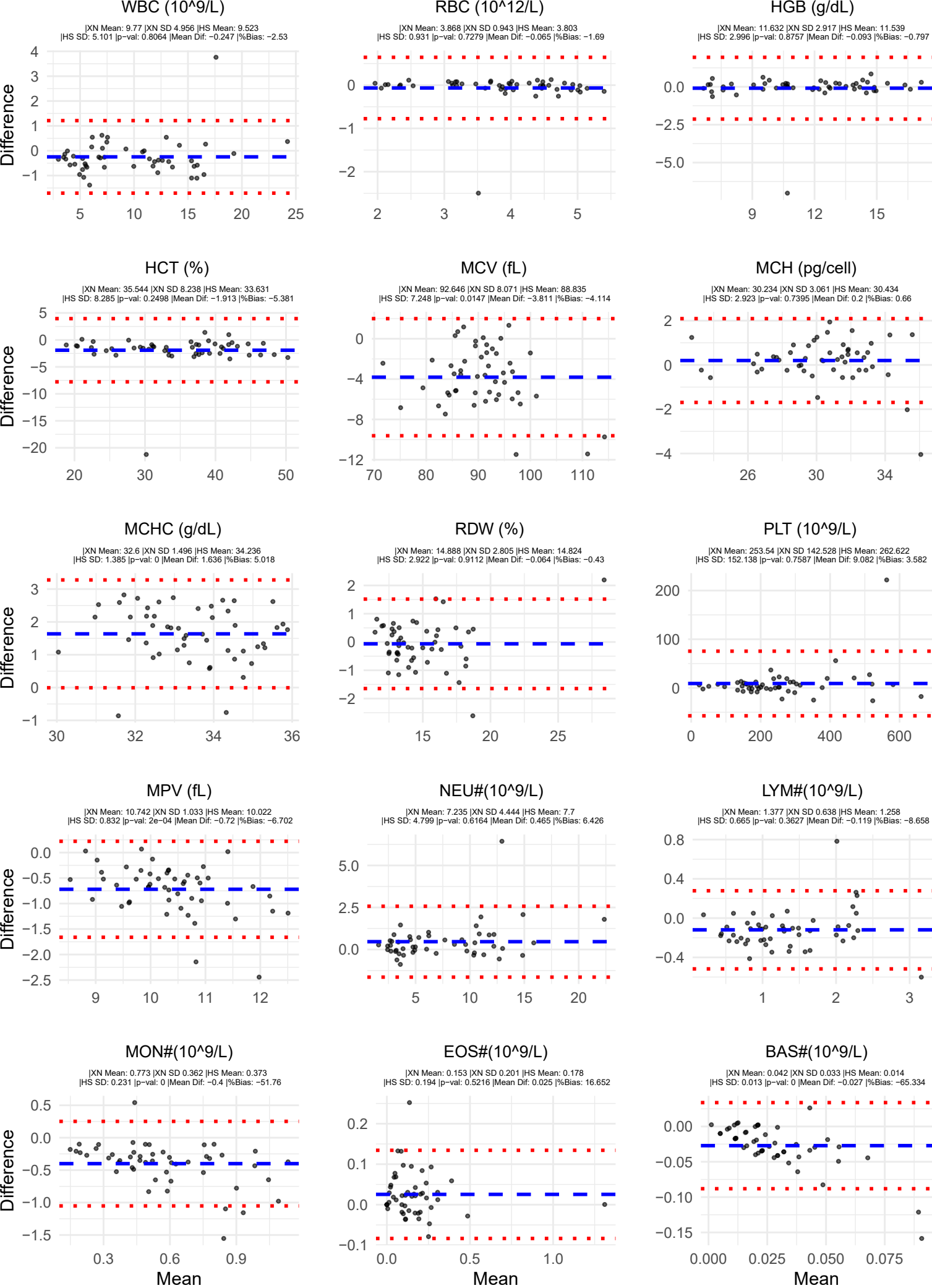

Supplement: Supplementary file 1 — Data S1: ijlh70032‐sup‐0001‐supinfo.zip. [file IJLH-48-353-s002.zip › Supplementary Material/Figure S5.pdf]

Figure S6 - Inpatient Scatter Plots

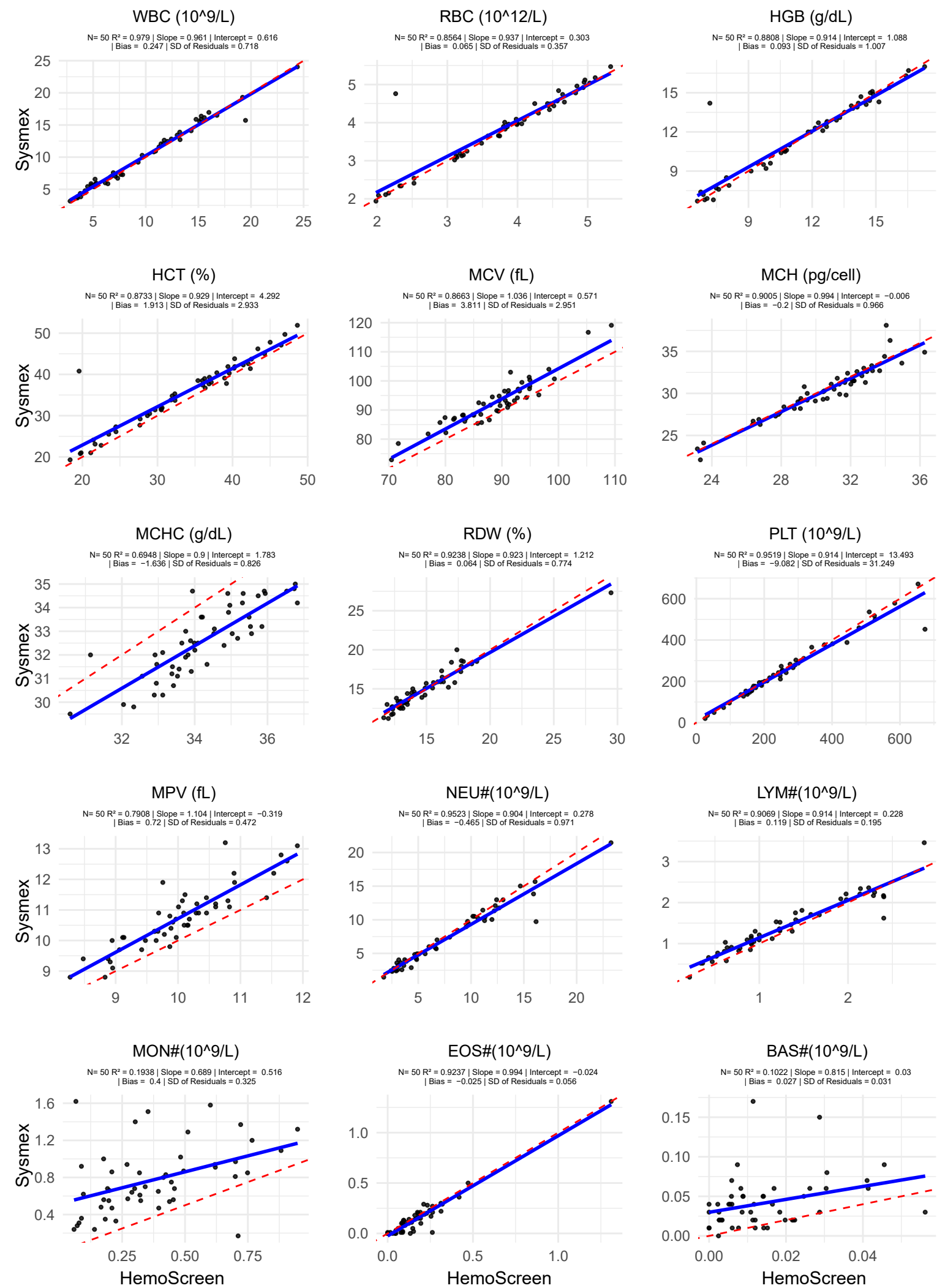

Supplement: Supplementary file 1 — Data S1: ijlh70032‐sup‐0001‐supinfo.zip. [file IJLH-48-353-s002.zip › Supplementary Material/Figure S6.pdf]

Figure S7 - Infusion Center Bland-Altman Plots

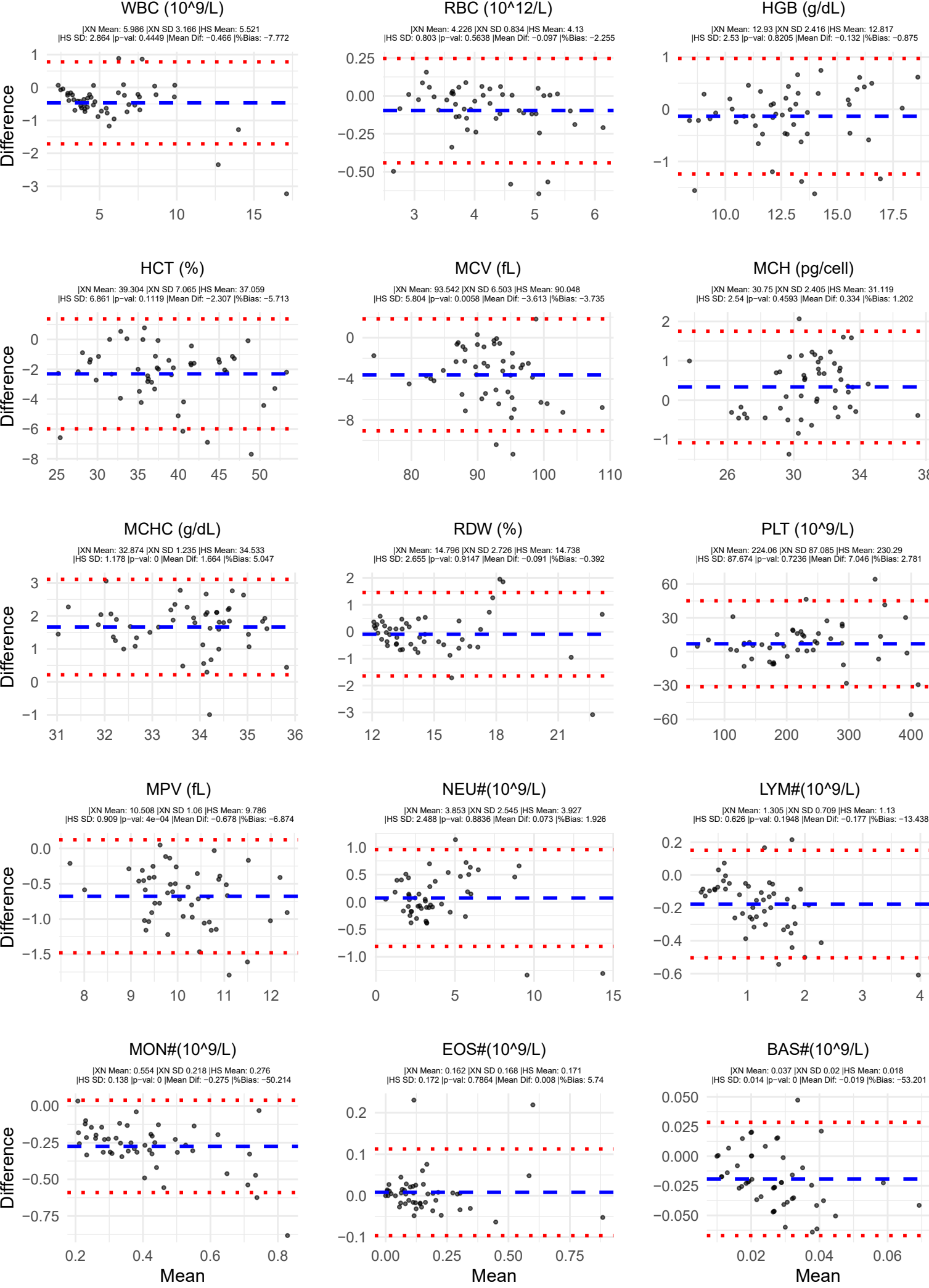

Supplement: Supplementary file 1 — Data S1: ijlh70032‐sup‐0001‐supinfo.zip. [file IJLH-48-353-s002.zip › Supplementary Material/Figure S7.pdf]

Figure S8 - Infusion Center Scatter Plots

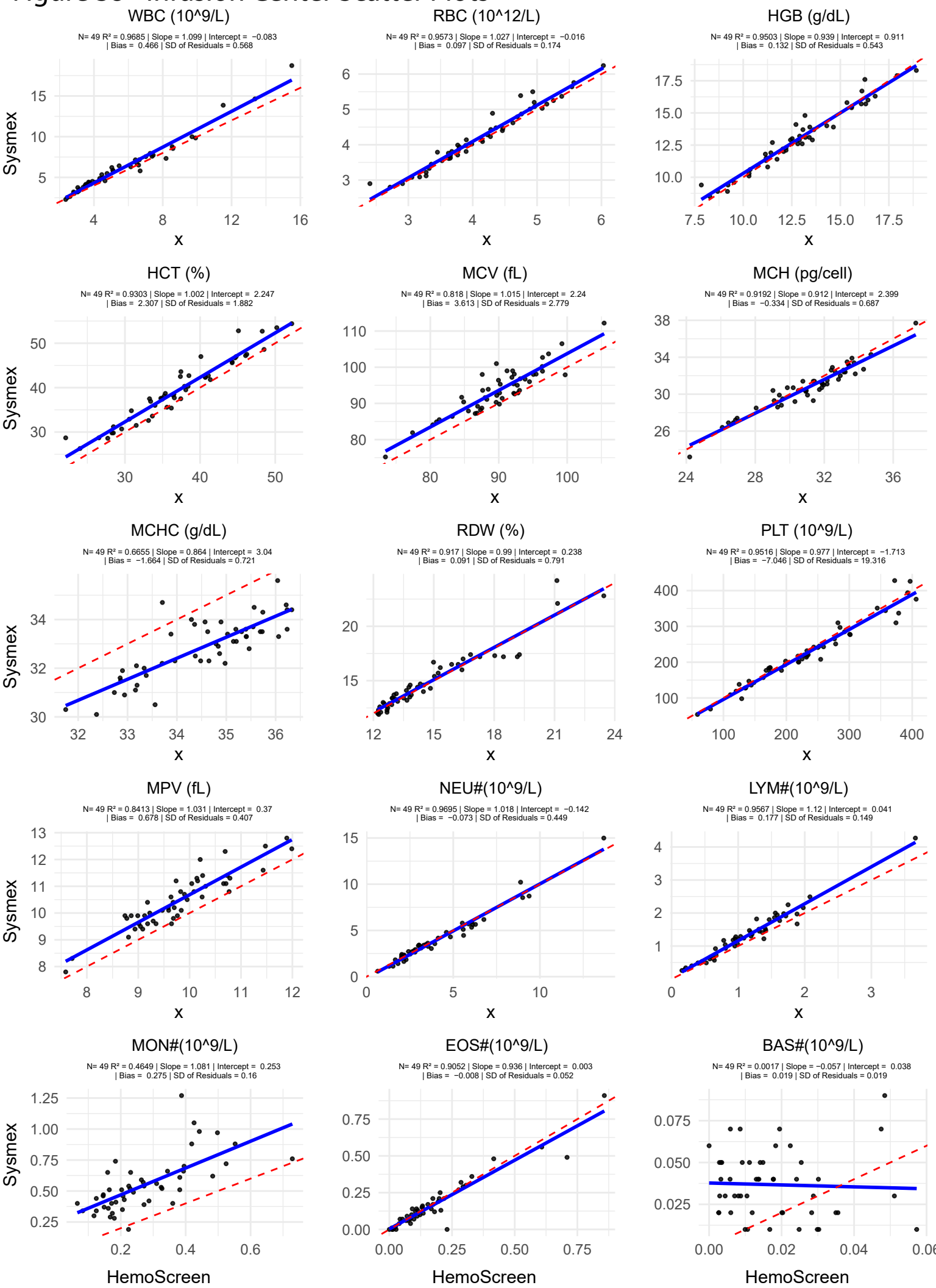

Supplement: Supplementary file 1 — Data S1: ijlh70032‐sup‐0001‐supinfo.zip. [file IJLH-48-353-s002.zip › Supplementary Material/Figure S8.pdf]
