# Supplementary material for: Clinical Evaluation of a Novel Point‐of‐Care Hematology Analyzer for Complete Blood Count With Differential
Source: Int J Lab Hematol. 2025 Dec 8;48(2):353–64. doi: 10.1111/ijlh.70032 (PMC12956494; doi:10.1111/ijlh.70032)
Supplement: Supplementary file 2 — Table S1: Between day imprecision on the HemoScreen instrument. Table S2: Hemoscreen flagging analysis. [file IJLH-48-353-s001.docx]

| **Table S1. Between Day Imprecision on the HemoScreen Instrument** | | | | |  |  |  |  |  |
| --- | --- | --- | --- | --- | --- | --- | --- | --- | --- |
|  |  |  |  |  |  |  |  |  |  |
|  |  | **Low** |  |  | **Medium** |  |  | **High** |  |
| **Parameter** | **Mean** | **SD** | **CV%** | **Mean** | **SD** | **CV%** | **Mean** | **SD** | **CV%** |
| WBC (10^9^/L) | 2.97 | 0.23 | 7.79 | 7.66 | 0.57 | 7.43 | 17.93 | 1.40 | 7.81 |
| RBC (10^12^/L) | 2.87 | 0.05 | 1.92 | 5.02 | 0.12 | 2.36 | 5.56 | 0.13 | 2.32 |
| HGB (g/dL) | 8.09 | 0.15 | 1.84 | 16.74 | 0.73 | 4.36 | 20.56 | 0.57 | 2.79 |
| HCT (%) | 19.80 | 0.44 | 2.21 | 39.76 | 1.71 | 4.30 | 48.19 | 1.41 | 2.93 |
| MCV (fL) | 68.96 | 0.61 | 0.89 | 79.18 | 2.12 | 2.68 | 86.64 | 0.70 | 0.81 |
| MCH (pg) | 28.17 | 0.34 | 1.20 | 33.34 | 0.85 | 2.55 | 36.97 | 0.34 | 0.93 |
| MCHC g/dL | 40.85 | 0.37 | 0.90 | 42.11 | 0.50 | 1.19 | 42.67 | 0.36 | 0.84 |
| RDW (%) | 14.41 | 0.95 | 6.59 | 15.18 | 0.60 | 3.97 | 13.15 | 0.23 | 1.75 |
| PLT (10^9^/L) | 72.65 | 2.04 | 2.81 | 247.38 | 11.03 | 4.46 | 556.26 | 26.59 | 4.78 |
| MPV (fL) | 9.46 | 0.11 | 1.12 | 9.58 | 0.09 | 0.93 | 9.79 | 0.12 | 1.20 |
| NEU# (10^9^/L) | 1.61 | 0.15 | 9.30 | 4.23 | 0.43 | 10.14 | 9.78 | 0.89 | 9.10 |
| LYM# (10^9^/L) | 1.03 | 0.11 | 10.85 | 2.67 | 0.22 | 8.07 | 6.12 | 0.66 | 10.79 |
| MON# (10^9^/L) | 0.21 | 0.04 | 18.26 | 0.49 | 0.04 | 7.91 | 1.29 | 0.13 | 10.43 |
| EOS# (10^9^/L) | 0.11 | 0.05 | 46.39 | 0.23 | 0.11 | 47.97 | 0.66 | 0.29 | 43.66 |
| BAS# (10^9^/L) | 0.01 | 0.00 | 10.76 | 0.04 | 0.00 | 10.42 | 0.09 | 0.01 | 9.07 |
| NEU% | 54.11 | 2.17 | 4.00 | 55.18 | 2.00 | 3.63 | 54.51 | 1.38 | 2.53 |
| LYM% | 34.48 | 1.40 | 4.06 | 34.85 | 0.93 | 2.67 | 34.05 | 1.13 | 3.31 |
| MON% | 6.97 | 1.10 | 15.72 | 6.37 | 0.68 | 10.69 | 7.17 | 0.39 | 5.51 |
| EOS% | 3.97 | 1.89 | 47.77 | 3.11 | 1.59 | 51.18 | 3.78 | 1.78 | 47.13 |
| BAS% | 0.47 | 0.03 | 5.59 | 0.49 | 0.02 | 3.99 | 0.49 | 0.01 | 2.61 |

| Table S2: Hemoscreen Flagging Analysis |  |  |  |  |  |
| --- | --- | --- | --- | --- | --- |
| HemoScreen flags |  |  |  |  |  |
| Flag(s) | Patient Type | | | | Total |
|  | ED | IP | OP | INF |  |
| Abnormal cells | 4 | 19 | 4 | 5 | 32 |
| Platelet clumps | 1 |  | 1 | 1 | 3 |
| Platelet clumps & abnormal cells |  | 2 | 2 |  | 4 |
| Platelet clumps & unreported WBC |  |  | 1 |  | 1 |
| Total | 5 | 21 | 8 | 6 | 40 |
|  |  |  |  |  |  |
| Platelet clumps flag and blood smear findings | |  |  |  |  |
| Blood smear findings | Patient Type | | | | Total |
|  | ED | IP | OP | INF |  |
| Platelet clumps | 1 |  | 1 | 1 | 3 |
| No platelet clumps |  | 2 | 3 |  | 5 |
| Total | 1 | 2 | 4 | 1 | 8 |
| Platelet clumps, % | 100% | 0% | 25% | 100% | 38% |
|  |  |  |  |  |  |
| Abnormal cells flag and blood smear findings | |  |  |  |  |
| Blood smear findings | Patient Type | | | | Total |
|  | ED | IP* | OP | INF |  |
| Abnormal cells | 1 | 9 | 1 | 3 | 14 |
| No abnormal cells | 3 | 8 | 3 | 2 | 16 |
| Total | 4 | 17 | 4 | 5 | 30 |
| Abnormal cells, % | 25% | 53% | 25% | 60% | 47% |
| *Two additional cases did not have blood smears made. |  |  |  |  |  |
|  |  |  |  |  |  |
| Comparison of HemoScreen and manual differentials | |  |  |  |  |
| Comparison | Patient Type | | | | Total |
|  | ED | IP* | OP** | INF |  |
| At least one significant difference*** | 1 | 9 | 2 | 3 | 15 |
| No significant differences | 4 | 10 | 5 | 3 | 22 |
| Total | 5 | 19 | 7 | 6 | 37 |
| Abnormal cells, % | 20% | 47% | 29% | 50% | 41% |
| *Two additional cases did not have blood smears made. |  |  |  |  |  |
| ** One additional case did not have a differential reported by the HemoScreen. |  |  |  |  |  |
| *** Significance level = 0.01 |  |  |  |  |  |
|  |  |  |  |  |  |
| Statistically significant differences between Hemoscreen and manual differentials | | | | |  |
| Difference(s) | Pt Type |  |  |  |  |
| NEU% 92.4 vs 78, MON% 3.9 vs 18 | ED |  |  |  |  |
| NEU% 83.4 vs 62, MON% 5.3 vs 17 | INF |  |  |  |  |
| NEU% 70.2 vs 52, LYM% 27.4 vs 41 | INF |  |  |  |  |
| NEU% 59.4 vs 34 | INF |  |  |  |  |
| NEU% 84.0 vs 70, MON% 1.3 vs 8 | IP |  |  |  |  |
| NEU% 81.5 vs 67, LYM% 11.1 vs 23 | IP |  |  |  |  |
| NEU% 76.3 vs 56, LYM% 8.9 vs 33 | IP |  |  |  |  |
| NEU% 62.1 vs 46, LYM% 29.3 vs 43 | IP |  |  |  |  |
| MON% 1.2 vs 10 | IP |  |  |  |  |
| MON% 0.4 vs 4 | IP |  |  |  |  |
| LYM% 7.0 vs 17 | IP |  |  |  |  |
| BAS% 0.6 vs 4 | IP |  |  |  |  |
| BAS% 0.2 vs 6 | IP |  |  |  |  |
| MON% 4.2 vs 17 | OP |  |  |  |  |
| MON% 3.9 vs 13 | OP |  |  |  |  |
